# Variation in mode of physical activity by ethnicity and time since immigration: a cross-sectional analysis

**DOI:** 10.1186/1479-5868-7-75

**Published:** 2010-10-14

**Authors:** Shilpa Dogra, Brad A Meisner, Chris I Ardern

**Affiliations:** 1 School of Recreation Management and Kinesiology, Acadia University, 550 Main St, Wolfville, NS. B4P-2R6, Canada; 2 School of Kinesiology and Health Science, 117 Bethune College, York University, 4700 Keele St., Toronto, ON, M3J 1P3, Canada; 3 School of Kinesiology and Health Science, 352 Bethune College, York University, 4700 Keele St., Toronto, ON, M3J 1P3, Canada

## Abstract

**Background:**

Physical activity (PA) levels are known to be significantly lower in ethnic minority and immigrant groups living in North America and Europe compared to the general population. While there has been an increase in the number of interventions targeting these groups, little is known about their preferred modes of PA.

**Methods:**

Using three cycles of the Canadian Community Health Survey (cycles 1.1, 2.1, 3.1; 2000-2005, n = 400,055) this investigation determined PA preferences by self-ascribed ethnicity (White, South Asian, South-East Asian, Blacks, Latin American, West Asian, Aboriginal persons and Other) and explored variation in PA preference across time since immigration categories (non-immigrant, established immigrant [> 10 years], and recent immigrant [≤ 10 years]). PA preferences over the past three months were collapsed into eight categories: walking, endurance, recreation, sports, conventional exercise, active commuting, and no PA. Logistic regression models were used to estimate the odds of participating in each PA across ethnicity and time since immigration compared to Whites and non-immigrants, respectively.

**Results:**

Compared to Whites, all other ethnic groups were more likely to report no PA and were less likely to engage in walking, with the exception of Aboriginal persons (OR: 1.25, CI: 1.16-1.34). Further, all ethnic groups including Aboriginal persons were less likely to engage in endurance, recreation, and sport activities, but more likely to have an active commute compared to Whites. Recent and established immigrants were more likely to have an active commute and no PA, but a lower likelihood of walking, sports, endurance, and recreation activities than non-immigrants.

**Conclusion:**

Ethnic minority groups and immigrants in Canada tend to participate in conventional forms of exercise compared to Whites and non-immigrants and are less likely to engage in endurance exercise, recreation activities, and sports. Health promotion initiatives targeting ethnic and immigrant groups at high-risk for physical inactivity and chronic disease should consider mode of PA preference in intervention development.

## Introduction

Immigrant populations in North America and Europe are healthier upon immigration compared to the general population, lending to a theory known as the *healthy immigrant effect *[1,2]. This has been attributed to higher education levels and health standards required for individuals to qualify for immigration to their host countries [3]. In recent years however, ethnic minorities in Canada and the United States have been found to be at particularly high risk for developing cardiometabolic diseases such as type 2 diabetes [4], cardiovascular disease [5], and metabolic syndrome [6].

In addition to this paradox of healthy immigrants and high-risk ethnicity, ethnic minorities, and new immigrant groups have consistently been shown to have lower physical activity (PA) levels than White or non-immigrant groups [7-11]. These differences may be related to socioeconomic status (SES) [12,13] and underemployment [14], or different social norms about PA [15,16]. New immigrants to Canada have been shown to have lower SES and longer work hours than non-immigrant groups [14], which leaves little time for PA or recreation activities. Moreover, the cost associated with certain activities can be an additional deterrent to adopting and maintaining an active lifestyle [17].

Canadian reports have indicated that physical inactivity is generally the only poor lifestyle habit exhibited by this population [18]. Taken together with a genetic predisposition for chronic disease among some ethnic minority groups, these lifestyle habits may be contributing to a compromised quality of life and a growing healthcare burden. Therefore, within an increasingly diverse society, it is essential that we understand the PA preferences of a range of ethnic and new immigrant populations so that appropriate and effective public health interventions and policy can be developed. Creating exercise programs or community programs with physical activities that are preferred by this cohort of Canadians may enhance the sustainability of such publicly funded programs. The purpose of this paper was therefore to determine which modes of PA were most prevalent among different ethnic groups and time-since-immigration groups.

## Methods

The Canadian Community Health Survey (CCHS) collects information related to health status, health care utilization, and health determinants. The CCHS is a nationally representative, population-based, cross-sectional survey. Three cycles (cycle 1.1, 2.1, and 3.1; 2000 to 2005, n = 400,055) of the CCHS were merged to ensure sufficient ethnic and time-since-immigration samples for valid comparisons. All data contained in the CCHS are self-reported and all respondents provided informed consent prior to participation. Detailed information on data collection methods and data weighting can be found in the CCHS user guide [19]. For purposes of the current analysis, the dataset was restricted to adults over the age of 20 years (n = 347,229) who had complete information for modes of PA (n = 301,418), ethnicity (n = 236,851), and time-since-immigration (n = 236, 596); the final sample size by sex was male = 105,984; female = 130,612. After exclusions, the prevalence of non-White ethnic and immigrant groups in the selected sample were within 1.2% of the original sample.

### Main Outcome and Exposure Variables

#### Ethnicity

Respondents self-reported their ethnicity into one of the 13 categories listed by the CCHS. For the purpose of comparison to previous Canadian studies [7,8], these groups were subsequently merged to form the following eight categories: White, South-Asian (East-Indian, Pakistani or Sri Lankan), South East (SE) Asian (i.e., SE Asian including Vietnamese, Cambodian, Indonesian and Laotian, or Chinese, Filipino, Japanese or Korean), Blacks, Latin American, West Asian (i.e., West Asian including Afghans and Iranians, or Arab descent or ancestry), Aboriginal persons, and Others.

#### Time-Since-Immigration

Respondents were asked in which country they were born. Those who responded with a country other than Canada were asked if they were born a Canadian citizen. From these questions, it was determined whether participants were Canadian or had immigrated to Canada. Respondents were also asked when they first came to Canada. This information was then used to create a variable with three categories, namely: non-immigrant, established immigrant (immigrated > 10 years ago) or recent immigrant (immigrated ≤ 10 years ago).

#### Physical Activities

Respondents were asked whether they participated in any of 21 physical activities over the past three months. As they were allowed to choose more than one activity, groups were not mutually exclusive. The physical activities were then merged to form the following groups: walking, endurance activities (i.e., swimming, running or jogging, cycling, or rollerblading), recreation activities (i.e., gardening, golfing, fishing, bowling, or dance), conventional exercise (i.e., home based exercise, aerobics classes, or weight training), sports (i.e., volleyball, basketball, ice hockey, ice skating, snowboarding or skiing, baseball, tennis, or soccer), active commuting (i.e., walking or cycling to work or school), no PA, or other physical activities. Walking was maintained as a separate category as it is a common PA among the general population. The remaining groupings were based on similarities of activities. Total metabolic equivalents were estimated based on the frequency and duration associated with each reported PA. A PA index was derived to classify participants as 'active' (> 3.0 kcal/kg/day), 'moderately active' (1.5-3.0 kcal/kg/day), or 'inactive' (< 1.5 kcal/kg/day) based on the Canadian Fitness and Lifestyle Research Institute cut-offs [20].

### Covariates and Demographic Variables

Self-reported height and weight were used to calculate body mass index (BMI; kg/m^2^) and participants were classified as underweight (< 18.5 kg/m^2^), normal weight (18.5-24.9 kg/m^2^), overweight (25-29.9 kg/m^2^), or obese (< 30 kg/m^2^). Age was categorized into the following four groups: 20-34 y, 35-49 y, 50-64 y, and ≥ 65 y. Highest level of education was self-reported as either grade school or some secondary school, secondary school graduate, some postsecondary education, or postsecondary graduate. Household income was categorized into 11 categories that ranged from no annual income to $80,000 (CDN) or more per year.

### Statistical Analyses

Pearson chi-square analyses and standardized adjusted residuals that denote deviations from a normal distribution were [21] calculated to determine differences in sample characteristics. Logistic regression analyses using each PA mode as an outcome with ethnicity and time-since-immigration as main exposures were performed. In accordance with previous research, these associations were assessed with adjustment for age alone and then again with age, sex, BMI, education, and income [7,8]. All analyses were conducted using SPSS version 17.0 with statistical significance set at alpha < 0.05. In order to compensate for the deliberate over-sampling of particular groups, population weights supplied by Statistics Canada were applied to the entire dataset to ensure accurate population estimates. To estimate variance, the sample population weights were re-scaled, standardized, and re-applied to the dataset.

## Results

Sample characteristics are shown in Table 1 in the form of percentages. Significant differences were noted between ethnic and time-since-immigration categories for all variables using the standardized adjusted residuals. The younger age categories were more prevalent among the ethnic minorities compared to Whites. On the other hand, recent immigrants tended to be in the younger age categories while established immigrants tended to be in the older age categories compared to non-immigrants. Generally, obesity prevalence was higher in Whites than any other ethnic minority group other than Aboriginal persons and Blacks. The majority of Aboriginal persons fell within the less than secondary education category, whereas the highest proportion of South-Asians and West-Asians fell within the post-secondary graduate category. With regards to income, recent immigrants had a lower prevalence in the high income categories compared to non-immigrants, while established immigrants had comparable incomes to non-immigrants. Consistent with previous Canadian research [7,8], non-White ethnic groups tended to have a higher proportion of individuals in the 'inactive' category, while recent immigrants had the highest proportion of 'inactive' individuals. The greatest variation was seen for recreation activities such that compared to ethnic minorities and immigrants, a larger proportion of Whites and non-immigrants self-reported participation in recreation activities. On the other hand the West Asian group had a similar prevalence of endurance, conventional exercise, sport and active commuting when compared to Whites (all p > 0.05). Lastly, recent immigrants had a similar prevalence of participation in endurance activities when compared to non-immigrants, while established immigrants had a similar prevalence of active commuting compared to non-immigrants. The "other" PA category was not assessed in subsequent regression models as all participants responded 'yes' to at least one of the three questions and as a result a binary comparison could not be made.

**Table 1:** Characteristics of sample by ethnicity and time since immigration

		Ethnicity								Time Since Immigration		
		
		White (n = 215,694)	South Asian (n = 3,284)	SE Asian (n = 4,962)	Black (n = 1,858)	Latin American (n = 861)	West Asian (n = 878)	Aboriginal persons (n = 6,921)	Others (n = 2,138)	Non-Immigrant (n = 204,764)	Established Immigrant (n = 25,616)	Recent Immigrant (n = 6,216)
**Age**	20-34	23.2	36.2	35.5	37.3	45.2	42.9	41.8	34.6	25.9	13.0	43.7
	
	35-49	30.8	38.3	36.2	36.4	39.2	35.1	31.5^	35	30.8	29.9	41.8
	
	50-64	24.5	18.2	18.2	19.4	11.2	17.7	17.7	19.1	23.6	30.9	10.6
	
	65+	21.5	7.3	10.1	6.9	4.4	4.2	9.0	11.3	19.6	26.2	3.8

**Sex**	Male	46.7	52.0	49.7	45.7^	44.4^	56.1	43.2	52.1	46.5	48.7	49.8
	
	Female	53.3	48.0	50.3	54.3^	55.6^	43.9	56.8	47.9	53.5	51.3	50.2

**Body Mass Index**	UW	2.3	5.2	9.0	2.6	3.2	3.4	1.7	2.5^	2.4	2.8	6.8
	
	Normal	44.9	56.2	70.0	45.9	45.4	49.8	33.6	47.3^	44.8	46.9	60.5
	
	OW	35.5	30.8	17.8	34.6	36.7	34.4^	34.2^	34.6^	34.9	36.4	25.5
	
	OB	17.2	7.8	3.2	16.9	14.7	12.4	30.4	15.6^	17.9	13.9	7.2

**Education**	> Secondary	24.1	16.5	14.1	18.4	16.9	14.5	45.0	18.4	24.1	22.0	11.9
	
	Secondary Graduate	18.0	16.5	21.9	15.5	22.0	15.4	12.5	17.1^	17.5	18.2	15.5
	
	Some post-secondary	7.4	9.3	8.1^	12.1	10.0	12.1	9.0	8.4^	8.0	6.1	8.2^
	
	Post-Secondary Graduate	50.5	57.7	55.9	54.0	51.1^	58.0	33.6	56.2	50.4	53.6	64.5

Income	> $5,000	0.9	1.3^	3.2	0.9^	3.7	3.2	2.7^	2.1^	0.9	0.9	3.9
	
	$5,000-14,999	9.6	4.1	6.5^	8.7^	8.9^	11.1^	20.6	8.1^	10.0	6.8	8.7
	
	$15,000-29,999	17.4	14.4	15.3^	22.9^	12.2	20.8^	22.7	19.3^	17.3	16.7^	19.7^
	
	$30,000-49,999	21.3	24.8^	21.8^	26.2	28.8^	28.2^	21.2^	18.9^	21.1	21.5^	25.4
	
	$50,000-$79,999	24.9	29.5	25.9^	22.4^	30.5	21.3^	19.3	24.1^	24.7	25.2^	25.4^
	
	$80,000 and more	25.9	25.8^	27.3^	18.9	16.0	15.4^	13.6	27.4^	26.1	28.9	16.9

**PA Index**	Active	21.3	16.1	17.9	17.8	19.1^	19.4^	22.7^	20.1^	22.6	20.8	16.9
	
	Moderate	24.6	18.4	20.8	18.8	20.8	16.8	20.5	22	24.9	22.9	19.7
	
	Inactive	54.1	65.5	61.3	63.4	60.1	63.8	56.8	57.9	52.5	56.3	63.4

**PA Mode**	Walking	68.3	56.7	55.8	55.3	57.5	56.2	70.8	59.5	69.1	64.2	55.7
	
	Endurance	34.6	29.7	35.8^	29.2	33.3^	33.6^	29.2	31.5	37.1	30.2	36.2^
	
	Recreation	60.0	38.8	40.0	37.1	38.0	30.5	51.7	48.4	62.4	51.0	35.7
	
	Exercise	36.6	45.0	43.7	45.8	44.1	38.5^	31.0	43.3	37.1	39.9	39.7
	
	Sports	28.8	24.3	27.6^	26.8^	27.4^	31.6^	25.0	29.9	26.9	18.8	28.4
	
	Commute	53.1	55.8	49.7	59.7	63.9	52.0^	60.5	52.1^	54.1	53.7^	59.3
	
	No PA	10.5	18.7	15.9	17.4	15.6	20.9	12.5	14.1	9.7	13.4	17.9

Note: All figures are in percentages and each coloumn sums up to 100% per variable, except for PA Mode. PA Mode figures represent % of sample who responded yes to participation in that particular activity. Sample size numbers are unweighted.

Abbreviations: SE: South-East; BMI: Body Mass Index; UW: underweight; OW: overweight; OB: obese; PA: Physical Activity.

Symbols: ^NS (*p *> 0.05) based on Pearson chi square analyses and standardized adjusted residuals.

Referent group for ethnicity analyses was 'White' and referent for time since immigration was 'Non-Immigrant'.

Table 2 provides odds ratios for the age adjusted models for modes of PA by ethnicity and time since immigration. The trend for walking, endurance, recreation, sports and no PA were similar between the South Asian, SE Asian and West Asian groups such that these groups were all *less *likely to participate in the aforementioned activities when compared to Whites. With the exception of active commuting, Blacks, Latin Americans and Others had similar trends such that they were *less *likely to participate in walking, endurance, recreation, and sports and *more *likely to participate in exercise compared to Whites. The time since immigration groups had similar trends with the exception of conventional exercise such that recent immigrants were *less *likely and established immigrants were *more *likely to engage in conventional exercise when compared to non-immigrants.

**Table 2:** Age adjusted odds ratios for modes of physical activity by ethnicity and immigration groups (by sex)

		Walking	Endurance	Recreation	Exercise	Sports	Active Commute	No PA
**A. Both Sexes**	White	1.00	1.00	1.00	1.00	1.00	1.00	1.00
	
	South Asian	0.58 (0.55-0.62)	0.57 (0.54-0.61)	0.37 (0.35-0.39)	1.23 (1.16-1.30)	0.51 (0.47-0.55)	1.04 (0.98-1.10)	2.52 (2.34-2.70)
	
	SE Asian	0.57 (0.54-0.60)	0.80 (0.76-0.84)	0.40 (0.38-0.42)	1.19 (1.13-1.25)	0.65 (0.60-0.70)	0.82 (0.78-0.86)	1.98 (1.85-2.13)
	
	Black	0.55 (0.51-0.60)	0.56 (0.52-0.62)	0.35 (0.32-0.38)	1.27 (1.17-1.38)	0.59 (0.53-0.66)	1.22 (1.13-1.33)	2.28 (2.05-2.54)
	
	Latin American	0.60 (0.54-0.68)	0.61 (0.54-0.69)	0.35 (0.32-0.40)	1.10 (0.99-1.24)	0.51 (0.44-0.59)	1.42 (1.26-1.60)	2.19 (1.87-2.56)
	
	West Asian	0.57 (0.51-0.64)	0.64 (0.57-0.72)	0.26 (0.23-0.29)	0.89 (0.80-1.00)	0.69 (0.60-0.80)	0.87 (0.78-0.98)	3.06 (2.66-3.51)
	
	Aboriginal persons	1.10 (1.03-1.17)	0.56 (0.52-0.59)	0.64 (0.61-0.68)	0.66 (0.62-0.70)	0.53 (0.49-0.58)	1.26 (1.19-1.34)	1.53 (1.40-1.67)
	
	Others	0.66 (0.61-0.72)	0.67 (0.61-0.73)	0.57 (0.53-0.62)	1.18 (1.09-1.29)	0.77 (0.69-0.86)	0.91 (0.84-0.99)	1.67 (1.48-1.89)
	
	Non-Immigrant	1.00	1.00	1.00	1.00	1.00	1.00	1.00
	
	Established Immigrant	0.80 (0.78-0.82)	0.88 (0.85-0.90)	0.64 (0.62-0.66)	1.26 (1.23-1.29)	0.81 (0.78-0.84)	1.03 (1.00-1.05)	1.30 (1.26-1.35)
	
	Recent Immigrant	0.55 (0.52-0.57)	0.65 (0.62-0.67)	0.30 (0.29-0.31)	0.91 (0.87-0.95)	0.66 (0.62-0.69)	1.13 (1.09-1.18)	2.68 (2.54-2.83)

**B.Males**	White	1.00	1.00	1.00	1.00	1.00	1.00	1.00
	
	South Asian	0.70 (0.65-0.75)	0.72 (0.67-0.79)	0.39 (0.36-0.42)	1.49 (1.38-1.61)	0.56 (0.51-0.62)	1.06 (0.99-1.15)	2.27 (2.04-2.51)
	
	SE Asian	0.65 (0.60-0.70)	0.84 (0.78-0.91)	0.41 (0.38-0.44)	1.27 (1.18-1.37)	0.70 (0.64-0.77)	0.85 (0.79-0.91)	2.00 (1.81-2.21)
	
	Black	0.60 (0.53-0.68)	0.75 (0.67-0.85)	0.35 (0.31-0.40)	1.54 (1.36-1.73)	0.80 (0.69-0.93)	1.23 (1.09-1.38)	1.93 (1.63-2.29)
	
	Latin American	0.79 (0.67-0.93)	0.77 (0.65-0.92)	0.33 (0.28-0.39)	1.30 (1.10-1.54)	0.66 (0.54-0.82)	1.32 (1.11-1.57)	1.72 (1.33-2.21)
	
	West Asian	0.71 (0.61-0.82)	0.77 (0.66-0.90)	0.22 (0.19-0.25)	1.08 (0.93-1.26)	0.98 (0.82-1.18)	0.93 (0.81-1.08)	2.78 (2.30-3.36)
	
	Aboriginal persons	1.15 (1.05-1.26)	0.61 (0.55-0.67)	0.66 (0.61-0.72)	0.77 (0.70-0.84)	0.57 (0.51-0.64)	1.26 (1.15-1.38)	1.46 (1.27-1.68)
	
	Others	0.68 (0.61-0.76)	0.68 (0.60-0.77)	0.53 (0.47-0.59)	1.04 (0.92-1.17)	0.82 (0.71-0.95)	0.83 (0.74-0.93)	1.80 (1.53-2.13)
	
	Non-Immigrant	1.00	1.00	1.00	1.00	1.00	1.00	1.00
	
	Established Immigrant	0.86 (0.83-0.89)	0.93 (0.89-0.97)	0.63 (0.60-0.65)	1.37 (1.32-1.43)	088 (0.83-0.92)	0.98 (0.95-1.02)	1.31 (1.24-1.39)_
	
	Recent Immigrant	0.62 (0.59-0.66)	0.73 (0.69-0.78)	0.31 (0.29-0.33)	0.95 (0.89-1.00)	0.78 (0.73-0.83)	1.18 (1.11-1.25)	2.45 (2.26-2.65)

**C. Females**	White	1.00	1.00	1.00	1.00	1.00	1.00	1.00
	
	South Asian	0.48 (0.44-0.52)	0.41 (0.37-0.45)	0.34 (0.31-0.37)	1.04 (0.96-1.12)	0.34 (0.29-0.39)	1.03 (0.95-1.11)	2.83 (2.56-3.13)
	
	SE Asian	0.49 (0.46-0.53)	0.75 (0.69-0.81)	0.38 (0.35-0.41)	1.13 (1.05-1.21)	0.56 (0.50-0.63)	0.80 (0.75-0.86)	2.00 (1.81-2.20)
	
	Black	0.49 (0.44-0.55)	0.42 (0.37-0.48)	0.35 (0.31-0.39)	1.08 (0.97-1.20)	0.30 (0.24-0.38)	1.21 (1.09-1.35)	2.56 (2.23-2.94)
	
	Latin American	0.44 (0.38-0.51)	0.50 (0.42-0.59)	0.38 (0.32-0.44)	0.95 (0.82-1.11)	0.40 (0.32-0.51)	1.49 (1.27-1.75	2.61 (2.14-3.19)
	
	West Asian	0.47 (0.40-0.56)	0.46 (0.38-0.56)	0.29 (0.25-0.35)	0.75 (0.63-0.89)	0.24 (0.17-0.33)	0.84 (0.71-0.99)	3.43 (2.80-4.20)
	
	Aboriginal persons	0.97 (0.89-1.06)	0.52 (0.48-0.57)	0.64 (0.59-0.69)	0.57 (0.53-0.62)	0.53 (0.47-0.60)	1.23 (1.14-1.33)	1.60 (1.42-1.79)
	
	Others	0.67 (0.59-0.77)	0.63 (0.55-0.73)	0.60 (0.53-0.68)	1.41 (1.25-1.59)	0.60 (0.49-0.73)	1.04 (0.92-1.17)	1.57 (1.31-1.87)
	
	Non-Immigrant	1.00	1.00	1.00	1.00	1.00	1.00	1.00
	
	Established Immigrant	0.76 (0.73-0.79)	0.81 (0.78-0.85)	0.63 (0.61-0.65)	1.19 (1.15-1.24)	0.66 (0.62-0.71)	1.08 (1.04-1.12)	1.32 (1.26-1.39)
	
	Recent Immigrant	0.46 (0.44-0.49)	0.56 (0.52-0.59)	0.28 (0.27-0.30)	0.89 (0.84-0.94)	0.48 (0.43-0.52)	1.10 (1.04-1.16)	2.94 (2.72-3.17)

Note: All odds ratios are adjusted for age.

Abbreviations: SE: South-east; PA: Physical Activity.

After adjusting for covariates, odds of PA varied such that all ethnic minorities were less likely to engage in walking, except for Aboriginal persons (OR: 1.25; CI: 1.16-1.34; Table 3A). All ethnic minorities were also less likely to engage in endurance, recreation, and sport activities compared to Whites. On the other hand, South Asian, SE Asian, Blacks, and Latin Americans were all more likely to engage in conventional forms of exercise compared to Whites; however, results for active commuting were more variable. Specifically, all Asian groups were *less *likely to have an active commute, whereas Blacks, Latin Americans, and Aboriginal persons were *more *likely to have an active commute compared to Whites. Taken together, all ethnic minorities were more likely to report no PA compared to Whites; however, some of these results were sex-specific as shown in Table 3B and 3C.

**Table 3:** Odds Ratios for modes of physical activity by ethnicity and sex

		Walking	Endurance	Recreation	Exercise	Sports	Active Commute	No PA
**A. Both Sexes**	White	1.00	1.00	1.00	1.00	1.00	1.00	1.00
	
	South Asian	0.67 (0.63-0.72)	0.58 (0.54-0.63)	0.52 (0.49-0.56)	1.27 (1.18-1.36)	0.51 (0.46-0.56)	0.91 (0.85-0.97)	2.08 (1.9-2.29)
	
	SE Asian	0.63 (0.59-0.67)	0.81 (0.76-0.87)	0.57 (0.54-0.61)	1.21 (1.14-.129)	0.72 (0.66-0.79)	0.70 (0.66-0.75)	1.69 (1.54-1.85)
	
	Black	0.62 (0.57-0.68)	0.64 (0.58-0.71)	0.48 (0.43-0.52)	1.38 (1.26-1.51)	0.70 (0.61-0.79)	1.15 (1.05-1.26)	1.79 (1.58-2.02)
	
	Latin American	0.77 (0.68-0.88)	0.71 (0.62-0.82)	0.54 (0.47-0.61)	1.33 (1.18-1.52)	0.63 (0.53-0.75)	1.15 (1.02-1.31)	1.40 (1.17-1.68)
	
	West Asian	0.70 (0.61-0.80)	0.78 (0.68-0.90)	0.41 (0.36-0.47)	0.97 (0.85-1.11)	0.92 (0.77-1.10)	0.76 (0.67-0.87)	2.24 (1.90-2.64)
	
	Aboriginal persons persons	1.25 (1.16-.1.34)	0.79 (0.73-0.85)	0.83 (0.78-0.88)	0.91 (0.85-0.98)	0.78 (0.71-0.86)	1.35 (1.27-1.44)	1.07 (0.97-1.19)
	
	Others	0.65 (0.59-0.72)	0.67 (0.61-0.75)	0.77 (0.70-0.84)	1.11 (1.00-1.22)	0.89 (0.78-1.00)	0.86 (0.78-0.94)	1.43-1.88)

**B. Males**	White	1.00	1.00	1.00	1.00	1.00	1.00	1.00
	
	South Asian	0.79 (0.72-0.86)	0.70 (0.64-0.76)	0.55 (0.50-0.60)	1.52 (1.38-1.67)	0.54 (0.48-0.61)	0.90 (0.82-0.99)	2.00 (1.75-2.82)
	
	SE Asian	0.77 (0.70-0.83)	0.86 (0.78-0.94)	0.61 (0.55-0.66)	1.36 (1.24-1.49)	0.79 (0.71-0.89)	0.74 (0.67-0.80)	1.63 (1.43-1.86)
	
	Black	0.67 (0.58-0.76)	0.89 (0.76-1.02)	0.51 (0.44-0.58)	1.71 (1.24-1.49)	0.90 (0.76-1.06)	1.16 (1.01-1.32)	1.57 (1.30-1.90)
	
	Latin American	1.03 (0.85-1.24)	0.96 (0.79-1.17)	0.55 (0.45-0.67)	1.51 (1.25-1.83)	0.91 (0.72-1.17)	1.01 (0.84-1.22)	1.00 (0.73-1.37)
	
	West Asian	0.85 (0.72-1.01)	0.97 (0.82-1.15)	0.37 (0.31-0.44)	1.20 (1.00-1.43)	1.21 (0.97-1.50)	0.83 (0.70-0.98)	2.26 (1.83-2.81)
	
	Aboriginal persons persons	1.28 (1.15-1.41)	0.83 (0.75-0.92)	0.83 (0.75-0.92)	1.07 (0.96-1.19)	0.83 (0.73-0.95)	1.30 (1.18-1.44)	0.99 (0.85-1.17)
	
	Others	0.68 (0.60-0.78)	0.67 (0.59-0.76)	0.66 (0.58-0.75)	1.00 (0.88-1.15)	0.87 (0.74-1.03)	0.77 (0.68-0.88)	1.56-2.24)

**C.Females**	White	1.00	1.00	1.00	1.00	1.00	1.00	1.00
	
	South Asian	0.57 (0.52-0.63)	0.44 (0.40-0.50)	0.48 (0.44-0.54)	1.08 (0.98-1.19)	0.36 (0.30-0.43)	0.93 (0.85-1.03)	2.18 (1.91-2.48)
	
	SE Asian	0.53 (0.49-0.59)	0.76 (0.69-0.84)	0.53 (0.49-0.58)	1.15 (1.05-1.26)	0.56 (0.50-0.66)	0.68 (0.63-0.75)	1.70 (1.50-1.94)
	
	Black	0.57 (0.50-0.65)	0.45 (0.39-0.53)	0.46 (0.41-0.53)	1.15 (1.02-1.31)	0.41 (0.32-0.52)	1.12 (0.99-1.27)	1.93 (1.64-2.27)
	
	Latin American	0.54 (0.46-0.64)	0.56 (0.47-0.68)	0.56 (0.47-0.66)	1.16 (0.97-1.37)	0.49 (0.37-0.65)	1.25 (1.05-1.49)	1.71 (1.36-2.15)
	
	West Asian	0.61 (0.49-0.75)	0.49 (0.39-0.53)	0.47 (0.37-0.58)	0.81 (0.65-1.00)	0.30 (0.20-0.45)	0.71 (0.58-0.87)	2.17 (1.68-2.80)
	
	Aboriginal persons persons	1.13 (1.02-1.25)	0.77 (0.69-0.85)	0.83 (0.76-0.91)	0.80 (0.73-0.88)	0.82 (0.71-0.94)	1.37 (1.26-1.50)	1.15 (1.00-1.31)
	
	Others	0.67 (0.57-0.77)	0.66 (0.56-0.78)	0.89 (0.77-1.03)	1.32 (1.14-1.52)	0.78 (0.62-0.98)	1.02 (0.88-1.18)	1.40 (1.14-1.71)

Note: All odds ratios are adjusted for age, sex, body mass index, income, and education.

Abbreviations: SE: South-east; PA: Physical Activity.

**Figure 1:**
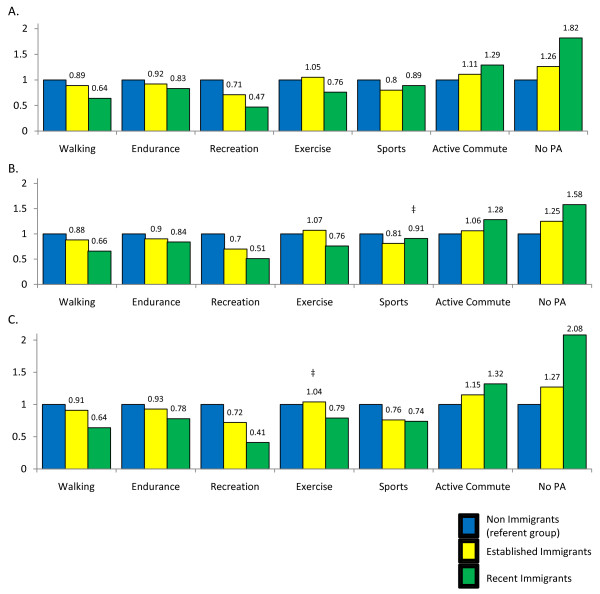
**Odds Ratios for modes of physical activity by time since immigration and sex**. A. Overall B. Males C. Females. ‡: not significant (p > 0.05); PA: physical activity

The odds for self-reporting different modes of PA across time-since-immigration categories varied significantly (Figure 1A). Those who immigrated to Canada were less likely to report walking, endurance, sports and recreation activities compared to non-immigrants, but *more *likely to report an active commute and no PA compared to non-immigrants. Recent immigrants were also less likely to engage in conventional forms of exercise (OR: 0.76; CI:0.71-0.81), while established immigrants (OR:1.05; CI:1.01-1.09) had similar exercise levels when compared to non-immigrants. Again, some of the associations varied by sex as indicated in Figure 1B and 1C.

The odds for reporting different modes of PA were generally similar between males and females of the same ethnic or immigrant group; however, some notable sex differences did occur. For example, compared to Whites, walking and endurance activity participation amongst West Asian and Latin Americans varied by sex. While Aboriginal males were similar to White males for conventional exercise, Aboriginal females were less likely to engage in this form of activity than White females (OR = 0.80, 0.73-0.88). Finally, compared to Whites, active commuting was less common amongst South-Asian, Blacks, and Other ethnic group men, whereas patterns amongst females in these groups did not differ.

The odds ratios for the various covariates from the adjusted model can be seen in Table 4. For education the trend was similar for all activities such that a higher education was associated with higher odds of participation. Income had a consistent association with no PA such that those whose household income was less than $100,000 per year were *less *likely to engage in activities compared to those whose household income was above $100,000 per year. Finally, those who were overweight or normal weight had higher odds of engaging in all activities, and were *less *likely to have no PA.

**Table 4:** Odds Ratios of covariates from fully adjusted model for modes of physical activity

		Walking	Endurance	Recreation	Exercise	Sports	Active Commute	No PA
**Education**	> secondary	0.59 (0.57-0.61)	0.51 (0.49-0.53)	0.74 (0.71-0.76)	0.50 (0.48-0.52)	0.52 (0.50-0.55)	0.79 (0.76-0.81)	2.16 (2.06-2.25)
	
	Secondary graduate	0.83 (0.80-0.61)	0.74 (0.72-0.76)	0.95 (0.92-0.98)	0.76 (0.74-0.78)	0.87 (0.83-0.90)	0.96 (0.94-0.99)	1.45 (1.38-1.52)
	
	Some Post-Secondary	0.88 (0.84-0.92)	0.98 (0.94-1.02)	1.07 (1.03-1.12)	0.98 (0.94-1.02)	1.09 (1.03-1.15)	1.17 (1.12-1.22)	1.13 (1.05-1.21)
	
	Post-Secondary Graduate	1.00	1.00	1.00	1.00	1.00	1.00	1.00

**Income**	No Income	0.72 (0.59-0.88)	0.67 (0.54-0.83)	0.54 (0.44-0.65)	0.48 (0.40-0.59)	0.95 (0.71-1.27)	0.88 (0.73-1.06)	2.60 (1.93-3.50)
	
	> 5 K	1.16 (1.00-1.34)	0.83 (0.72-0.96)	0.74 (.065-0.85)	0.45 (0.39-0.52)	0.62 (0.51-0.75)	0.93 (0.81-1.06)	2.22 (1.75-2.81)
	
	5-9.9 K	0.96 (0.87-1.05)	0.59 (0.54-0.65)	0.39 (0.36-0.42)	0.39 (0.35-0.43)	0.36 (0.32-0.42)	0.98 (0.90-1.06)	3.85 (3.27-4.53)
	
	10-14.9 K	0.85 (0.79-0.92)	0.57 (0.53-0.62)	0.50 (0.46-0.54)	0.43 (0.40-0.47)	0.47 (0.43-0.52)	0.93 (0.87-1.00)	3.57 (3.07-4.15)
	
	15-19.9 K	0.87 (0.80-0.94)	0.60 (0.55-0.65)	0.60 (0.56-0.65)	0.44 (0.41-0.47)	0.48 (0.43-0.53)	0.96 (0.90-1.03)	3.33 (2.86-3.87)
	
	20-29.9 K	0.84 (0.78-0.90)	0.67 (0.63-0.72)	0.73 (0.68-0.78)	0.45 (0.43-0.48)	0.54 (0.50-0.59)	0.92 (0.87-0.98)	2.96 (2.56-3.43)
	
	30-39.9 K	0.82 (0.76-0.88)	0.75 (0.70-0.80)	0.85 (0.80-0.91)	0.46 (0.44-0.50)	0.66 (0.61-0.71)	0.90 (0.84-0.95)	2.66 (2.30-3.08)
	
	40-49.9 K	0.80 (0.75-0.86)	0.82 (0.76-0.87)	0.92 (0.87-0.99)	0.51 (0.48-0.54)	0.66 (0.62-0.72)	0.87 (0.82-0.93)	2.56 (2.20-3.00)
	
	50-59.9 K	0.86 (0.81-0.93)	0.91 (0.86-0.98)	1.02 (0.95-1.09)	0.52 (0.48-0.55)	0.76 (0.70-0.82)	0.88 (0.83-0.94)	2.22 (1.91-2.58)
	
	60-79.9 K	0.87 (0.82-0.93)	0.94 (0.89-1.00)	1.09 (1.02-1.16)	0.56 (0.53-0.60)	0.90 (0.84-0.96)	0.88 (0.83-0.94)	1,90 (1.64-2.20)
	
	80-100.K	0.92 (0.86-0.98)	1.27 (1.20-1.35)	1.28 (1.20-1.36)	0.72 (0.68-0.76)	1.30 (1.22-1.38)	0.84 (0.79-0.89)	1.38 (1.20-1.60)
	
	100 K or more	1.00	1.00	1.00	1.00	1.00	1.00	1.00

**BMI**	Underweight	1.00 (0.93-1.08)	1.18 (1.09-1.28)	0.74 (0.68-0.79)	1.00 (0.93-1.09)	1.13 (1.01-1.26)	1.11 (1.04-1.19)	1.32 (1.20-1.45)
	
	Normal	1.21 (1.17-1.25)	1.57 (1.52-1.62)	1.06 (1.03-1.09)	1.35 (1.31-1.40)	1.69 (1.61-1.77)	1.10 (1.07-1.13)	0.69 (0.66-0.73)
	
	Overweight	1.12 (1.08-1.15)	1.45 (1.39-1.50)	1.17 (1.14-1.21)	1.22 (1.18-1.26)	1.78 (1.69-1.87)	1.03 (1.00-1.06)	0.72 (0.69-0.76)
	
	Obese	1.00	1.00	1.00	1.00	1.00	1.00	1.00

Note: All odds ratios are from the adjusted regression models used for the corresponding PA modes

## Discussion

Using a multi-year, cross-sectional, nationally representative database, we sought to characterize the PA preferences of the Canadian population of ethnic minorities and immigrants. Our primary finding was that, with the exception of Aboriginal groups, all ethnic minorities and immigrant groups were more likely to engage in conventional forms of exercise such as home-based exercise, aerobics, and weight training, when compared to Whites and non-immigrants, respectively. It was also found that all ethnic and immigrant groups were less likely to engage in walking, endurance exercise and recreation activities and were more likely to be physically inactive. These findings have implications for interventions targeting ethnic and immigrant Canadians, as most research has focused on culturally relevant activities such as tai-chi for Chinese or yoga for South-Asians, when conventional exercise seems to be a preferred activity.

### Modes of Physical Activity by Ethnicity

The finding that ethnic minority groups in Canada are less physically active than Whites is consistent with previous research [7]. However, the finding that all of these groups are more likely to engage in conventional forms of exercise such as aerobics, weight training, and home-based exercise is novel. Previous research has generally focused on *overall *PA levels rather than the specific modes of PA [7-12]. To our knowledge few studies to date have analyzed PA modes among ethnic minorities or immigrant groups. One of these was a study conducted in a group of older Canadian adults consisting of mainly Vietnamese, Cambodian, Polish, and Latin-American immigrants [22]. In this study, walking was the most frequently reported PA among Latin Americans, followed by aerobics. Amongst the Polish sample the most prevalent activity was yard and house work. This study did not have a White comparison group and the sample size was small (n = 61), limiting direct comparison to the current investigation. Second, Floyd et al. [13] analyzed the different activity preferences of African-Americans and Hispanic-Americans using U.S. census data from 1984 (n = 1469; terminology related to ethnicity has been preserved from the paper being referenced). Results indicated that two types of activities differed by ethnic background. Sport activities (bowling, basketball, and baseball) and associated-social activities (church activities, clubs, voluntary organizations, and parties) were reported more frequently by Blacks than Whites, leading the authors to conclude that there were more similarities than differences among the activity preferences of Whites versus Blacks in that sample. This is in contrast to the current findings which indicate that sports, exercise, and recreation activities were significantly different between Whites and all other ethnic groups, including Blacks and Latin Americans. These differences may be attributed to time (i.e. changes in PA patterns over the past decade), differences in composition of ethnic minorities in Canada and the United States, or differences in reporting methods. For instance, Floyd et al. asked participants to rank their preferred activities while the CCHS questionnaire prompts participants to list activities in which they engage.

A more recent study conducted by Chiang et al. [23] used focus groups and a qualitative analysis to gain an understanding of culturally preferred exercise programs in older adults who were immigrants of East-Asian descent, Spanish speaking immigrants, and Native Americans. In their sample, walking was again the preferred mode of PA among all groups. Other activities mentioned were stretching and tai-chi in the Cantonese group and dance and socializing in the Latino group. On this basis, the authors also concluded that there were more similarities than differences among ethnic groups. The present study found similar trends among ethnic groups but only in comparison to Whites. Therefore, these data support the finding that among ethnic minorities there are more similarities; however, when compared to Whites, there are distinct differences in PA preferences.

### Physical Activity Modes by Time-Since-Immigration

While previous research has indicated lower PA levels among immigrants in Canada [8], to our knowledge, no population-based studies have assessed the variation of PA modes by time-since-immigration. The Canadian Fitness and Lifestyle Research Institute have reported rates of sport participation among Canadian immigrants; however, they did not include other modes of PA such as exercise, recreation or commute related PA. Nonetheless, these data suggest that individuals born in Canada are more likely to participate in sport than individuals born outside of Canada. Self-reported prevalence of sport participation was 35-40% amongst the Canadian-born sample. In contrast, those who immigrated before 1990 had participation rates of only 20% and recent immigrants had participation rates of approximately 25% [24]. Our results are consistent with this report such that recent immigrants had a higher prevalence of sport participation compared to established immigrants; however, both groups were less likely to participate in sports than non-immigrants.

The odds for engaging in walking, endurance, recreation and conventional exercise were lowest among recent immigrants, followed by established immigrants. Although a temporal relationship can not be inferred from these data, these findings suggest a degree of acculturation among immigrants in Canada to the extent that the longer one lives in the country, the more likely they are to report higher levels of PA. Acculturation to PA participation and dietary habits of United States Asian and Hispanic immigrants has been documented [25]. Additionally, research assessing the healthy immigrant effect in Canada indicates that the gap between immigrants and non-immigrants for risk of chronic diseases related to active lifestyles begins to narrow with time spent in the country [1], suggesting the same acculturation effect we observed. Although we did not assess chronic disease specifically, a similar and consistent narrowing gap between the various modes of PA (as seen in Table 1) between recent immigrants, established immigrants and non-immigrants was observed. Moreover, this finding is similar to that of Tremblay et al. [8] who found that the prevalence of total leisure-time activity (> 3.0 kcal/kg/day) is higher amongst longer-term immigrants in Canada.

Finally, it is important to mention that some sex differences were observed in our study. Previous research indicates that women are less likely to engage in PA in general, and that women in all ethnic groups are less active than males [7]. Similarly, lower levels of participation in sports have been consistently noted when comparing Canadian males to females [26]. Overall, males and females of the same ethnic or immigrant group had similar PA patterns in our study. However, there were noticeable differences among the West Asian group such that males had similar patterns for walking, endurance, conventional exercise, sports and active commuting as White males, while females were less likely to participate in all activities and were more likely to report no PA than White females.

### Implications

Based on our findings, it is evident that interventions targeting ethnically diverse and immigrant Canadians should focus on conventional exercise as opposed to walking, sport, or endurance activities. An example of such an approach is the *Exercise on Prescription *program in the Netherlands which is currently assessing the impact of this program on a group of ethnically diverse women from low SES communities [27]. This program consists of 20 sessions of supervised PA and assistance with organizing participation in various exercise activities such as aerobics, aqua-aerobics, dance, and sports. Based on the current results, this is precisely the type of program that may be preferred by this segment of the population; however, in order to create a culturally relevant delivery method and to optimize participation and adherence rates, PA interventions must be developed in consultation with members of each community. Previous research has shown that success of interventions depends on consideration of socio-cultural norms, attitudes, and beliefs [28]. Additionally, as demonstrated in a recent study of insulin sensitivity in older Chinese adults, more conventional PA modes (e.g., resistance training) may lead to greater health benefits than some culturally specific exercises such as tai-chi [29].

### Limitations and Strengths

There are some limitations to this analysis that should be noted. As the CCHS contains self-reported data it is susceptible to social desirability and misclassification bias. We also did not have information available on culturally-specific physical activities such as tai-chi or yoga as these preferences would have been captured as part of the "other activities" reported. Given that the CCHS is a cross-sectional survey, it is not possible to exclude the fact that differences between recent and established immigrants could be attributed in part to differential bias in PA reporting, the era of immigration [24], acculturation to social norms of PA, or other unmeasured factors [1]. Finally, as the CCHS only assessed leisure-time PA, differences in occupational activity are unknown and may account for some of the observed differences in total energy expenditure by ethnicity and time-since-immigration. However, there are also several strengths to this analysis. First, we pooled three cycles of the CCHS, which produced a large sample size of ethnic minorities and immigrants. Second, we included a range of age groups in this analysis which produced generalizable results for adults over the age of 20 years, while previous research has focused primarily on ethnically diverse samples of older adults.

## Conclusion

In summary, ethnic minority groups and new immigrants in Canada were found to be less physically active and engage in more conventional forms of exercise such as aerobics, weight training and home-based exercise than Whites and non-immigrants. We conclude that while mode of PA participation seems to be similar among ethnic minorities and immigrant groups, they vary considerably from that of Whites and non-immigrant groups. Future research should explore whether or not culturally relevant modes of delivery for conventional forms of PA leads to greater adherence to programs and, ultimately, a greater impact on optimizing health.

## Abbreviations

BMI: Body Mass Index; CCHS: Canadian Community Health Survey; CI: Confidence Interval; OR: Odds Ratio; PA: Physical Activity; SE: South-East; SES: Socioeconomic Status; SPSS: Statistical Package for the Social Sciences

## Competing interests

The authors declare that they have no competing interests.

## Authors' contributions

SD conceived the idea, conducted the analysis and drafted the manuscript; BAM participated in the design of the study and assisted with drafting the manuscript; CIA assisted with statistical analyses and manuscript preparation. All authors read and approved the final version of the manuscript.
